# Association Between Succinate Receptor SUCNR1 Expression and Immune Infiltrates in Ovarian Cancer

**DOI:** 10.3389/fmolb.2020.00150

**Published:** 2020-08-31

**Authors:** Jiawen Zhang, Qinyi Zhang, Yongbin Yang, Qingying Wang

**Affiliations:** ^1^Department of Obstetrics and Gynecology, Shanghai Tenth People’s Hospital, School of Medicine, Tongji University, Shanghai, China; ^2^Department of Obstetrics and Gynecology, Shanghai General Hospital, Shanghai Jiao Tong University, Shanghai, China

**Keywords:** SUCNR1, ovarian cancer, immune infiltration, T cell exhaustion, survival

## Abstract

**Background:**

The activation of succinate receptor 1 (SUCNR1) by extracellular succinate has been found to regulate immune cell function. However, the clinical significance of SUCNR1 in ovarian cancer and its correlation with tumor-infiltrating lymphocytes remain unclear.

**Methods:**

The genetic alteration and expression patterns of SUCNR1 were analyzed by using cBioPortal and Gene Expression Omnibus (GEO) datasets. Kaplan-Meier Plotter was used to assess the prognostic value of SUCNR1 in patients with ovarian cancer. The correlations between SUCNR1 expression and immune infiltration, gene markers of immune cells, cytokines, chemokines, or T cell exhaustion were explored by using TIMER and TISIDB platforms. We also performed Gene Set Enrichment Analysis (GSEA) to reveal biological function of SUCNR1 in ovarian cancer.

**Results:**

The expression of SUCNR1 was closely related to tumor infiltrating lymphocytes, multiple gene markers of immune cells, and T cell exhaustion in ovarian cancer. The expression of SUCNR1 was also associated with the expression of cytokine- or chemokine-related genes. Moreover, GSEA revealed that various immune-related pathways might be regulated by SUCNR1. In addition, we found that SUCNR1 was amplified in ovarian cancer, and the high expression of SUCNR1 predicted worse progression-free survival (*p* = 0.0073, HR = 1.49, 95% CI = 1.11–2).

**Conclusion:**

These results highlight the role of SUCNR1 in regulating tumor immunity in ovarian cancer.

## Introduction

Accumulating evidences indicate that tumor microenvironment (TME) and immune cell infiltration play critical roles in the development and treatment of cancers (Anandappa et al., 2020; Galon and Bruni, 2020). The metabolic stress imposed by the immunosuppressive TME on infiltrating immune cells can lead to tumor immune escape (Li et al., 2019b). Within the TME, soluble metabolites released by cancer cells not only activate their own oncogenic signaling pathways to promote tumor growth and metastasis, but also change the surrounding immune cells to accelerate tumor progression (Huang and Mellor, 2014; Ngwa et al., 2019). As an important intermediate of the tricarboxylic acid cycle, succinate can induce inflammatory cytokines (IL-1β, IL-6, IL-8, and TNF-α) (Tannahill et al., 2013; Park et al., 2018; Li et al., 2019a). It has also been demonstrated as a signal for the process involved in various pathological statuses, such as ischemia, hypoxia, metabolic disorders, and cancers (Tretter et al., 2016; Jiang and Yan, 2017; Kula-Alwar et al., 2019).

Extracellular succinate function is achieved by binding its specific G protein-coupled receptor succinate receptor 1 (SUCNR1/GPR91) (He et al., 2004). SUCNR1 is highly expressed in kidney, heart, liver, retinal, and immune cells (Cho, 2018). Previous studies have reported that SUCNR1 can orchestrate the expansion of T-helper 17 cell (Th17) population, regulate mast cell activation, mediate release of IL-1β, and guide dendritic cells into the lymph nodes, thus playing a critical role in immune-mediated arthritis (Littlewood-Evans et al., 2016; Rubić-Schneider et al., 2017; Saraiva et al., 2018). Keiran et al. (2019) reported that SUCNR1 signaling regulated the phenotype of adipose-tissue-resident macrophages (ATMs) and served as a link between metabolism and inflammation. The *Sucnr1*^–/–^ mice also showed impaired migration of dendritic cells (Rubic et al., 2008), a reduced number of macrophage (van Diepen et al., 2017), and weakened immune responses to streptomycin and polyethylene glycol treatment (Lei et al., 2018). However, the expression pattern of SUCNR1 in cancers and its role in tumor immunity remain largely elusive.

Ovarian cancer is one of the most malignant cancers in the female reproductive system. It is estimated that there are more than 290,000 new cases and 180,000 deaths each year worldwide (Bray et al., 2018). Nowadays, it has been confirmed that tumor-related immune modulation plays a critical role in controlling the fate of ovarian cancer cells, prognosis, and treatment (Drakes and Stiff, 2018; Lee et al., 2019). In the current study, we explored the potential role and clinical significance of SUCNR1 in ovarian cancer from multiple databases. We found that the high expression of SUCNR1 was not only closely correlated with the infiltration level of various immune cells, but also associated with T cell exhaustion and diverse immune markers. These findings may provide a novel strategy for ovarian cancer immunotherapy by targeting SUCNR1.

## Materials and Methods

### Bioinformatic Analysis

The TISIDB platform^1^ was used to analyze the correlation of SUCNR1 expression with tumor-infiltrating lymphocytes, including activated CD8^+^ T cells, effector memory CD8^+^ T cells, activated CD4^+^ T cells, effector memory CD4^+^ T cells, regulatory T cells, natural killer cells, neutrophils, macrophages, activated dendritic cells, activated B cells, and myeloid-derived suppressor cells (MDSC) (Ru et al., 2019). We also used TISIDB to analyze the association between the expression of succinate-related regulators and molecular subtypes in ovarian cancer.

The TIMER^2^ and TIMER2.0^3^ platforms were used to investigate the association between SUCNR1 expression and the infiltration level of B cells, CD4^+^ T cells, CD8^+^ T cells, neutrophils, macrophages, and dendritic cells (Li et al., 2016, 2017). The correlation of the genetic status of SUCNR1 with the immune infiltration level of immune cells, correlation of the mutant status of MUC16 with the immune infiltration level of immune cells, and correlation of the mutation status of specific genes (MUC16, TP53, PTEN, KRAS, BRCA1, BRCA2, and PIK3CA) with the expression of SUCNR1 were analyzed by TIMER2.0. In addition, correlation of SUCNR1 expression with various gene markers of immune cells, T cell exhaustion, cytokines, and chemokines were explored via TIMER correlation module. Moreover, the clinical relevance of tumor immune subsets and SUCNR1 expression were explored using the TIMER outcome module.

The cBioPortal platform^4^ was used to analyze the genetic alteration of SUCNR1 across different cancer types (Cerami et al., 2012; Gao et al., 2013).

Gene Expression Omnibus (GEO) datasets^5^ were used to evaluate the expression of SUCNR1 between 12 ovarian surface epithelial cells and 12 laser capture microdissected serous ovarian cancers (GSE14407), and to explore the differential gene expression in visceral adipose tissue from *Sucnr1*^*flox/flox*^ and LysM-*Sucnr1*^–/–^ mice (GSE120121). We used GSE9891 (including 285 ovarian cancer samples) to analyze the correlation of SUCNR1 expression with markers of immune cells, exhausted T cells, cytokines and chemokines in ovarian cancer. We also used GSE39204 to analyze the expression of SUCNR1 in 30 serous ovarian cancer samples, 13 endometrioid ovarian cancer samples and 16 clear cell cancer samples.

The Kaplan-Meier plotter platform^6^ was used to assess the prognostic value of SUCNR1 in patients with ovarian cancer (Nagy et al., 2018). Overall survival (OS) and progression-free survival (PFS) of patients with high and low expression levels of SUCNR1 were displayed using Kaplan-Meier survival curves.

The LinkedOmics platform^7^ was used to perform the Gene Set Enrichment Analysis (GSEA) to explore the biological functions and immune-related pathways potentially regulated by SUCNR1 in ovarian cancer (Vasaikar et al., 2018).

### Statistical Analysis

The correlation of SUCNR1 expression with immune infiltration level was determined by the TIMER and TISIDB platforms using Spearman’s correlation analysis. The correlation of gene expressions were analyzed by using Spearman’s correlation analysis. The association between the expressions of succinate-related regulators and molecular subtypes in ovarian cancer was assessed by the TISIDB platform using the Kruskal-Wallis Test. Survival rates were assessed using Kaplan-Meier curves and the log-rank test. Student’s *t* test was used to compare the expression level of different samples in GEO datasets. The data were calculated by GraphPad^TM^ software (version 6.01, GraphPad Software, Inc., United States) and presented as mean ± SD. *p*-values <0.05 were considered statistically significant.

## Results

### The Expression of SUCNR1 Is Correlated With Immune Infiltration in Ovarian Cancer

**FIGURE 1:**
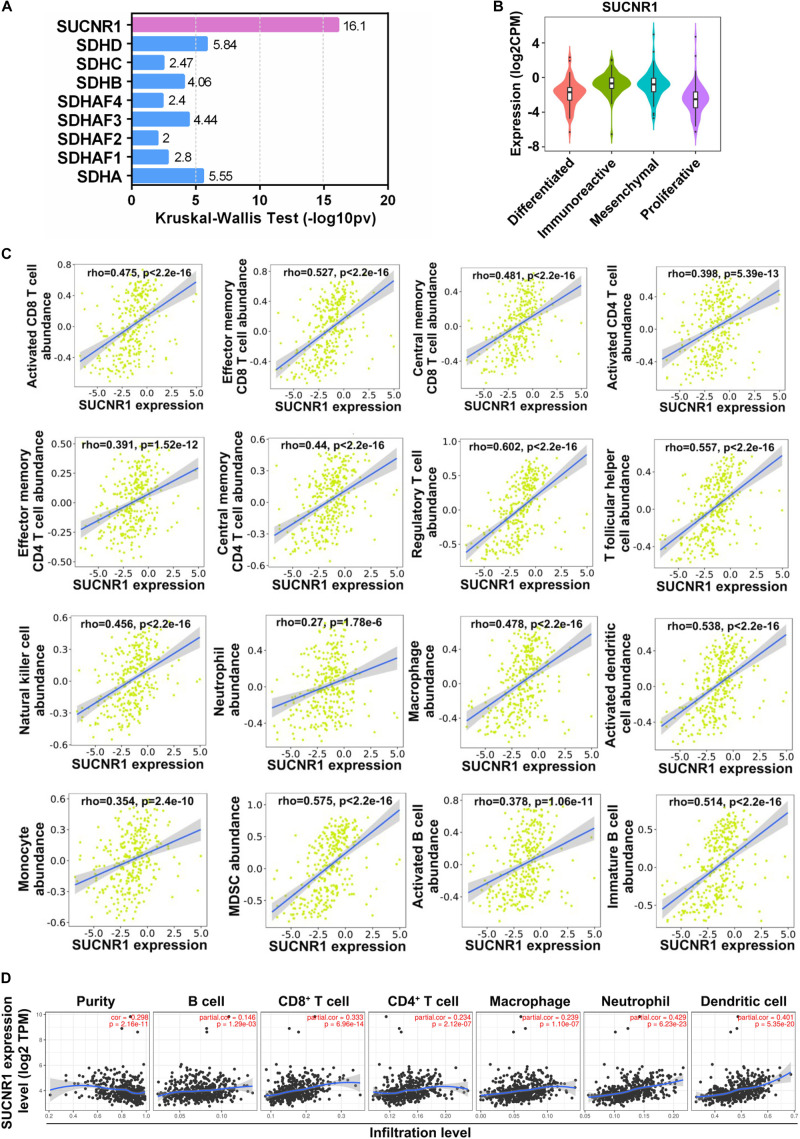
The expression of SUCNR1 is correlated with immune infiltration in ovarian cancer. **(A)** Association between nine succinate-related regulators with molecular subtypes of ovarian cancer available on the TISIDB database. Kruskal-Wallis Test values were shown. **(B)** Association between the expression of SUCNR1 with molecular subtypes of ovarian cancer available on the TISIDB database. **(C)** Correlation between the expression of SUCNR1 and the abundance of different tumor infiltrating lymphocytes, including activated CD8^+^ T cells, effector memory CD8^+^ T cells, central memory CD8^+^ T cells, activated CD4^+^ T cells, effector memory CD4^+^ T cells, central memory CD4^+^ T cells, regulatory T cells, T follicular helper cells, NK (natural killer cells), neutrophils, macrophages, activated dendritic cells, monocytes, MDSC (myeloid-derived suppressor cells), activated B cells, and immature B cells, in ovarian cancer are available on the TISIDB database. **(D)** Correlation of SUCNR1 expression with infiltrating levels of B cells, CD8^+^ T cells, CD4^+^ T cells, macrophages, neutrophils, and dendritic cells in ovarian cancer are available on the TIMER database. Correlation coefficients (rho values) and *p* values were shown.

To explore whether succinate-related regulators were involved in the infiltration of immune cells in ovarian cancer, we first investigated the association between nine succinate-related regulators with molecular subtypes of ovarian cancer by using the TISIDB platform. As shown in Figures 1A,B, SUCNR1 was the most relevant factor for the immunoreactive phenotype of ovarian cancer. Next, we demonstrated that the expression of SUCNR1 was significantly associated with the abundance of immune cells, such as activated CD8^+^ T cells (rho = 0.475, *p* < 2.2e-16), effector memory CD8^+^ T cells (rho = 0.527, *p* < 2.2e-16), activated CD4^+^ T cells (rho = 0.398, *p* = 5.13e-13), effector memory CD4^+^ T cells (rho = 0.391, *p* = 1.52e-12), regulatory T cells (rho = 0.602, *p* < 2.2e-16), natural killer cells (rho = 0.456, *p* < 2.2e-16), neutrophils (rho = 0.27, *p* = 1.78e-6), macrophages (rho = 0.478, *p* < 2.2e-16), activated dendritic cells (rho = 0.475, *p* < 2.2e-16), activated B cells (rho = 0.378, *p* = 1.06e-11), and MDSC (rho = 0.575, *p* < 2.2e-16), in ovarian cancer (Figure 1C). Moreover, the correlation between the expression of SUCNR1 and immune cell infiltration was also confirmed by TIMER, XCELL, and CIBERSORT tools (Figure 1D and Supplementary Figure S1).

### The Expression of SUCNR1 Is Correlated With Immune Cell Markers

**FIGURE 2:**
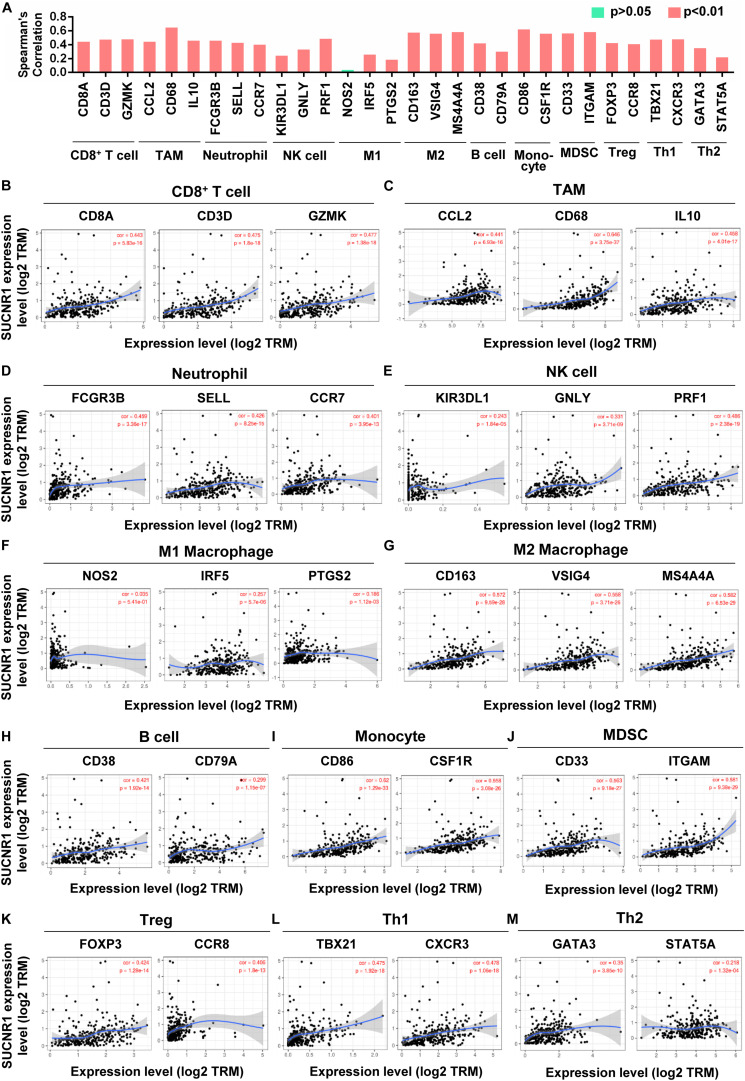
The expression of SUCNR1 is correlated with immune cell markers in ovarian cancer. Markers include CD8A, CD3D, and GZMK of CD8^+^ T cells; CCL2, CD68, and IL10 of TAM (tumor-associated macrophages); FCGR3B, SELL, and CCR7 of neutrophils; KIR3DL1, GNLY, and PRF1 of NK (natural killer cells); NOS2, IRF5, and PTGS2 of M1 macrophages; CD163, VSIG4, and MS4A4A of M2 macrophages; CD38 and CD79A of B cells; CD86 and CSF1R of monocytes; CD33 and ITGAM of MDSC (myeloid-derived suppressor cells); FOXP3 and CCR8 of Treg (regulatory T cells); TBX21 and CXCR3 of Th1 (T-helper 1 cells); GATA3 and STAT5A of Th2 (T-helper 2 cells). **(A)** The summary of the correlation between SUCNR1 and immune cell markers. **(B–M)** Correlation of SUCNR1 expression with immune gene makers of CD8^+^ T cells **(B)**, TAM **(C)**, neutrophils **(D)**, NK cells **(E)**, M1 macrophages **(F)**, M2 macrophages **(G)**, B cells **(H)**, monocytes **(I)**, MDSC **(J)**, Treg **(K)**, Th1 **(L),** and Th2 **(M)** in ovarian cancer are available on the TIMER database. Correlation coefficients (rho values) and *p* values were shown.

We evaluated the relationship between SUCNR1 and diverse immune infiltrating cells. The correlation between SUCNR1 expression and immune markers of multiple immune cells were analyzed using the TIMER platform. The results demonstrated that the expression level of SUCNR1 was significantly correlated with most markers of different subsets of immune cells. For instance, CD8^+^ T cell markers (such as CD8A, CD3D, and GZMK, *p* < 0.0001), neutrophil markers (such as FCGR3B, SELL, and CCR7, *p* < 0.0001), natural killer cell markers (such as KIR3DL1, GNLY, and PRF1, *p* < 0.0001), tumor-associated macrophage (TAM) markers (such as CCL2, CD68, and IL10, *p* < 0.0001) and M2 macrophage markers (such as CD163, VSIG4, and MS4A4A, *p* < 0.0001) showed significant correlation with the expression of SUCNR1 (Figures 2A–J). We also revealed significant association of SUCNR1 with immune markers of regulatory T cells (Treg) (FOXP3 and CCR8, *p* < 0.0001), T-helper 1 cells (Th1) (TBX21 and CXCR3, *p* < 0.0001), and T-helper 2 cells (Th2) (GATA3 and STAT5A, *p* < 0.0001) (Figures 2A,K–M). Moreover, these findings were confirmed by the GSE9891 database (Supplementary Figure S2). Together, this data strongly suggests that SUCNR1 may function as an immune infiltration regulator in ovarian cancer.

### The Expression of SUCNR1 Is Associated With T Cell Exhaustion

**FIGURE 3:**
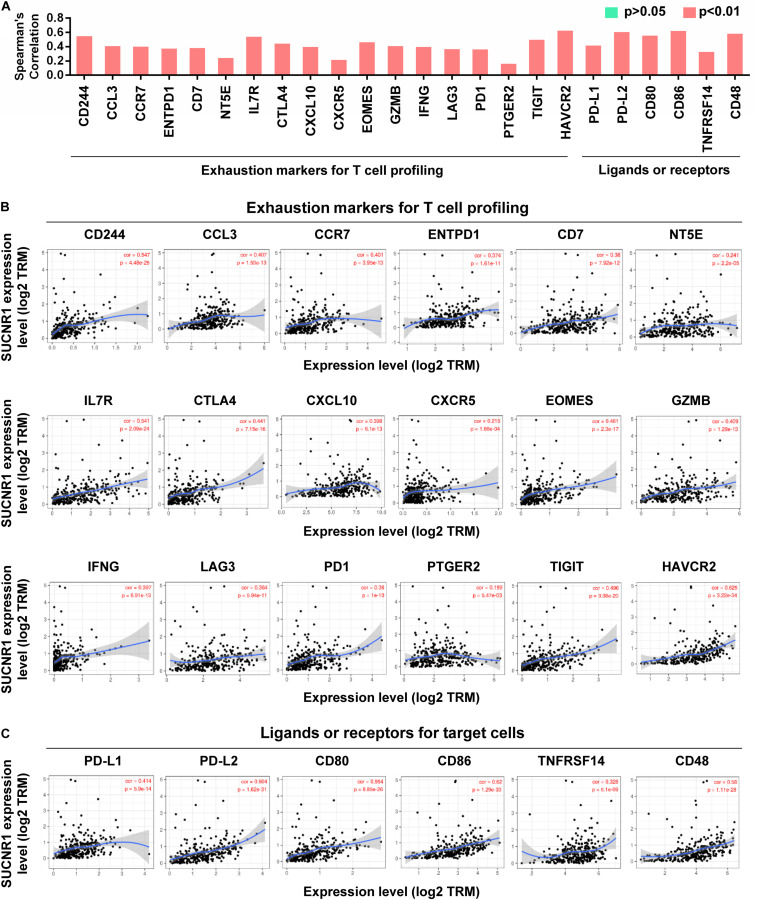
The expression of SUCNR1 is associated with T cell exhaustion in ovarian cancer. **(A)** The summary of the correlation between SUCNR1 and T cell exhaustion markers. **(B)** Correlation of SUCNR1 expression with exhaustion markers for T cells, including CD244, CCL3, CCR7, ENTPD1, CD7, NT5E, IL7R, CTLA4, CXCL10, CXCR5, EOMES, GZMB, IFNG, LAG3, PD1, PTGER2, TIGIT, and HAVCR2, in ovarian cancer are available on the TIMER database. **(C)** Correlation of SUCNR1 expression with ligands or receptors, including PD-L1, PD-L2, CD80, CD86, TNFRSF14, and CD48, in ovarian cancer are available at the TIMER database. Correlation coefficients (rho values) and *p* values were shown.

The exhaustion of T cells is often associated with inefficient control of tumor growth and metastasis (Wherry and Kurachi, 2015). We assessed the relationship between SUCNR1 expression and phenotypic markers of exhausted T cells by using the TIMER database. Interestingly, exhaustion markers of T cells, including CD244 (cor = 0.547, *p* = 4.48e-25), CCL3 (cor = 0.407, *p* = 1.53e-13), ENTPD1 (cor = 0.374, *p* = 1.61e-11), CTLA4 (cor = 0.441, *p* = 7.15e-16), EOMES (cor = 0.461, *p* = 2.3e-17), LAG3 (cor = 0.364, *p* = 5.94e-11), PD1 (cor = 0.36, *p* = 1e-10), TIGIT (cor = 0.496, *p* = 2.08e-20), and HAVCR2 (cor = 0.625, *p* = 3.22e-34), were significantly correlated with the expression of SUCNR1 (Figures 3A,B). Moreover, SUCNR1 expression was also significantly associated with other ligands or receptors for inhibitory pathways, such as PD-L1 (cor = 0.414, *p* = 5.9e-14), PD-L2 (cor = 0.604, *p* = 1.62e-31), CD48 (cor = 0.58, *p* = 1.11e-28), CD80 (cor = 0.554, *p* = 8.85e-26), CD86 (cor = 0.62, *p* = 1.29e-33), and TNFRSF14 (cor = 0.328, *p* = 5.1e-9) (Figures 3A,C). Moreover, the correlation between SUCNR1 and T cell exhaustion was confirmed by the GSE9891 database (Supplementary Figure S2). These findings suggest that SUCNR1 may regulate T cell exhaustion.

### Correlation Analysis of SUCNR1 Expression With Cytokines and Chemokines

**FIGURE 4:**
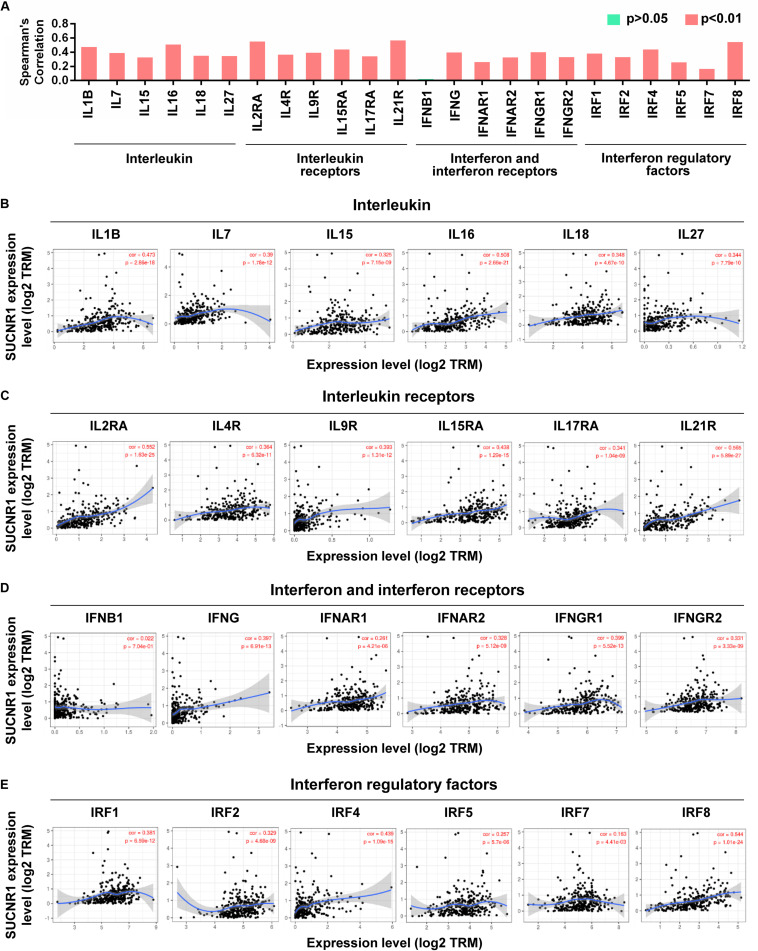
The expression of SUCNR1 is associated with cytokines in ovarian cancer. **(A)** The summary of the correlation between SUCNR1 and cytokines. **(B–E)** Correlation of SUCNR1 expression with interleukin, including IL1B, IL7, IL15, IL16, IL18, and IL27 **(B)**, interleukin receptors, including IL2RA, IL4R, IL9R, IL15RA, IL17RA, and IL21R **(C)**, interferon and interferon receptors, including IFNB1, IFNG, IFNAR1, IFNAR2, IFNGR1, and IFNGR2 **(D),** and interferon regulatory factors, including IRF1, IRF2, IRF4, IRF6, IRF7, and IRF8 **(E)**, in ovarian cancer are available on the TIMER database. Correlation coefficients (rho values) and *p* values were shown.

**FIGURE 5:**
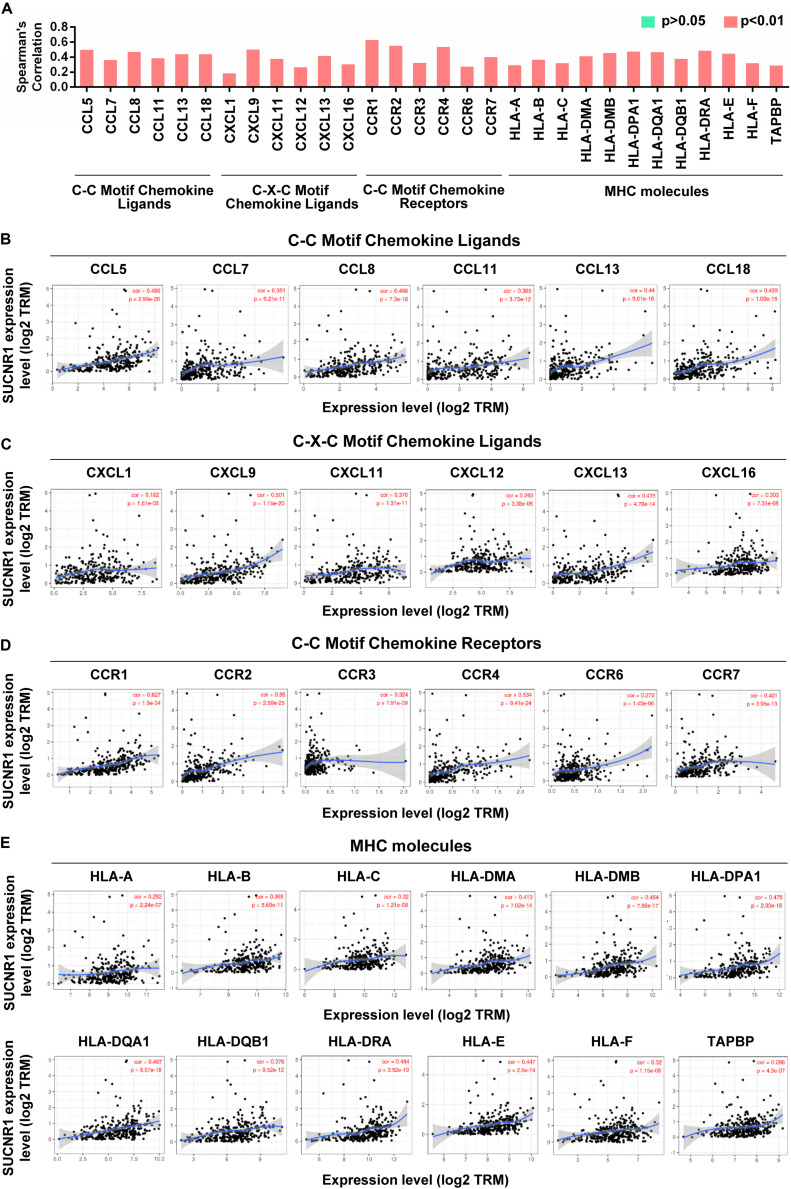
The expression of SUCNR1 is associated with chemokines and MHC molecules in ovarian cancer. **(A)** The summary of the correlation of SUCNR1 expression with chemokines and MHC molecules. **(B–D)** Correlation of SUCNR1 expression with C-C motif chemokine ligands, including CCL5, CLL7, CCL8, CCL11, CCL13, and CCL18 **(B)**, C-X-C motif chemokine ligands, including CXCL1, CXCL9, CXCL11, CXCL12, CXCL13, and CXCL16 **(C)**, and C-C motif chemokine receptors, including CCR1, CCR2, CCR3, CCR4, CCR6, and CCR7 **(D)**, in ovarian cancer are available on the TIMER database. **(E)** Correlation of SUCNR1 expression with MHC molecules, including HLA-A, HLA-B, HLA-C, HLA-DMA, HLA-DMB, HLA-DPA1, HLA-DQA1, HLA-DQB1, HLA-DRA, HLA-E, HLA-F, and TAPBP, in ovarian cancer are available on the TIMER database. Correlation coefficients (rho values) and *p* values were shown.

Cytokines and chemokines are important for controlling immune cell infiltration (Koizumi et al., 2007; Fridman et al., 2012). We observed the association of SUCNR1 expression with interleukins, including IL1B (cor = 0.473, *p* = 2.86e-18), IL7 (cor = 0.39, *p* = 1.78e-12), IL16 (cor = 0.508, *p* = 2.66e-21), and IL18 (cor = 0.348, *p* = 4.67e-10), in ovarian cancer (Figures 4A,B). The expression of SUCNR1 also showed significant correlation with the expressions of interleukin receptors (such as IL2RA, IL9R, and IL15RA, *p* < 0.0001), interferon receptors (such as IFNAR1, IFNAR2, IFNGR1, and IFNGR2, *p* < 0.0001), and even interferon regulatory factors (such as IRF1, IRF2, IRF4, and IRF5, *p* < 0.0001), in ovarian cancer (Figures 4A,C–E). Moreover, we found that the expressions of C-C motif chemokine ligands (such as CCL5, CCL7, CCL8, and CCL11, *p* < 0.0001), C-X-C motif chemokine ligands (such as CXCL9, CXCL11, CXCL12, and CXCL13, *p* < 0.0001) and C-C motif chemokine receptors (such as CCR1, CCR2, CCR4, and CCR7, *p* < 0.0001) had a strong correlation with SUCNR1 expression (Figures 5A–D). Furthermore, the differential gene expression in visceral adipose tissue from *Sucnr1*^*flox/flox*^ and LysM-*Sucnr1*^–/–^ mice were also analyzed based on the GEO database. As shown in Supplementary Figures S4A–D, several cytokine- or chemokine-related genes (such as *Cxcl10*, *Cxcl12*, *Ccl*2, *Ccl5*, *Il9*, *Il11*, *Irf1*, and *Irf3*, *p* < 0.05) were downregulated in *Sucnr1*^–/–^ tissues (GSE120121). Besides, MHC molecules, including HLA-B (cor = 0.365, *p* = 5.69e-11), HLA-DMA (cor = 0.413, *p* = 7.02e-14), HLA-DPA1 (cor = 0.475, *p* = 2.03e-18), HLA-DQA1 (cor = 0.467, *p* = 8.07e-18), and HLA-E (cor = 0.447, *p* = 2.6e-16), were significantly correlated with the expression of SUCNR1 in ovarian cancer (Figures 5A,E). Additionally, these findings were also confirmed by the GSE9891 database (Supplementary Figure S3).

### The Genetic Profile and Prognostic Value of SUCNR1 in Ovarian Cancer

**FIGURE 6:**
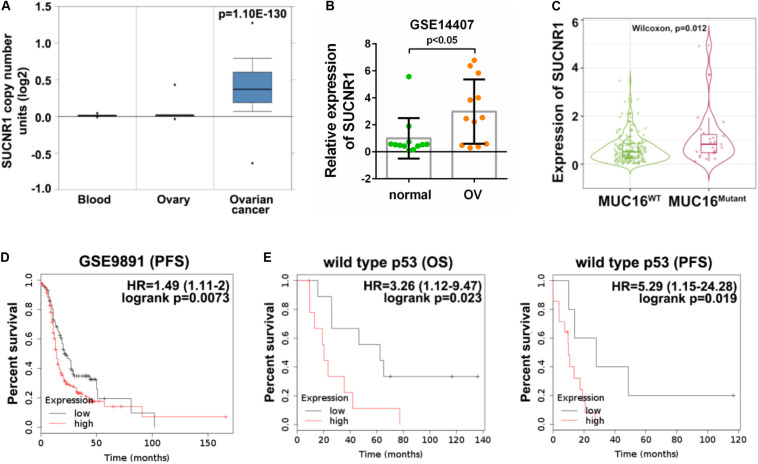
The genetic profile and prognostic value of SUCNR1 in ovarian cancer. **(A)** Copy number of SUCNR1 in normal blood, ovary, and ovarian cancer are available on the Oncomine database. **(B)** The expression of SUCNR1 between ovarian surface epithelial cells and serous ovarian cancer available at GSE14407, ^∗^*p* < 0.05. **(C)** The expression of SUCNR1 between MUC16 wild-type samples and MUC16 mutant samples of ovarian cancer are available on the TIMER2.0 database, ^∗^*p* < 0.05. **(D)** Kaplan-Meier PFS (progression-free survival) curves in SUCNR1 high and low expression ovarian cancer patients from GSE9891. **(E)** Kaplan-Meier OS (overall survival) and PFS curves in SUCNR1 high and low expression ovarian cancer patients with wild-type p53 from GSE9891.

We evaluated the clinical implication of SUCNR1 in ovarian cancer. According to cBioPortal, SUCNR1 showed a higher percentage of amplification in several cancer types, including ovarian cancer (Supplementary Figure S5A). The Oncomine database demonstrated that the copy number of SUCNR1 was significantly elevated in ovarian cancer than in normal blood and ovary samples (Figure 6A). We assessed the expression of SUCNR1 between ovarian cancer and normal tissues by using the GEO database. Compared to healthy ovarian surface epithelia (OSE), SUCNR1 was upregulated in serous ovarian cancer epithelia (GSE14407, Figure 6B). However, there was no difference in the expression of SUCNR1 between serous and endometrioid ovarian cancer, while the expression of SUCNR1 in clear cell carcinoma was lower than that in serous carcinomas (GSE39204, Supplementary Figure S5B). Intriguingly, the expression of SUCNR1 was higher in mutated MUC16/CA125 samples than in wild-type MUC16 samples (Figure 6C). Meanwhile, mutated MUC16/CA125 was also correlated with immune cell infiltration (Supplementary Figure S6). However, SUCNR1 expression was not dysregulated in mutated TP53, PTEN, KRAS, BRCA1, BRCA2, and PIK3CA samples (Supplementary Figure S5C).

The predictive value of SUCNR1 for prognosis in ovarian cancer was analyzed by the Kaplan-Meier plotter database. These results demonstrated that a high expression of SUCNR1 was associated with poor PFS (*p* = 0.0073, HR = 1.49, 95% CI = 1.11–2), but not with OS (*p* = 0.34, HR = 1.21, 95% CI = 0.82–1.79), in ovarian cancer patients (GSE9891, Figure 6D and Supplementary Figure S5D). Moreover, SUCNR1 was also a risk prognostic factor with HR > 1 for both OS and PFS in patients with wild-type TP53, but not with mutated TP53 (GSE9891, Figure 6E and Supplementary Figure S5E). Together, this data suggests that dysregulated expression of SUCNR1 is related to the clinical outcome for ovarian cancer.

### The Amplification of SUCNR1 Was Correlated With Immune Infiltration

**FIGURE 7:**
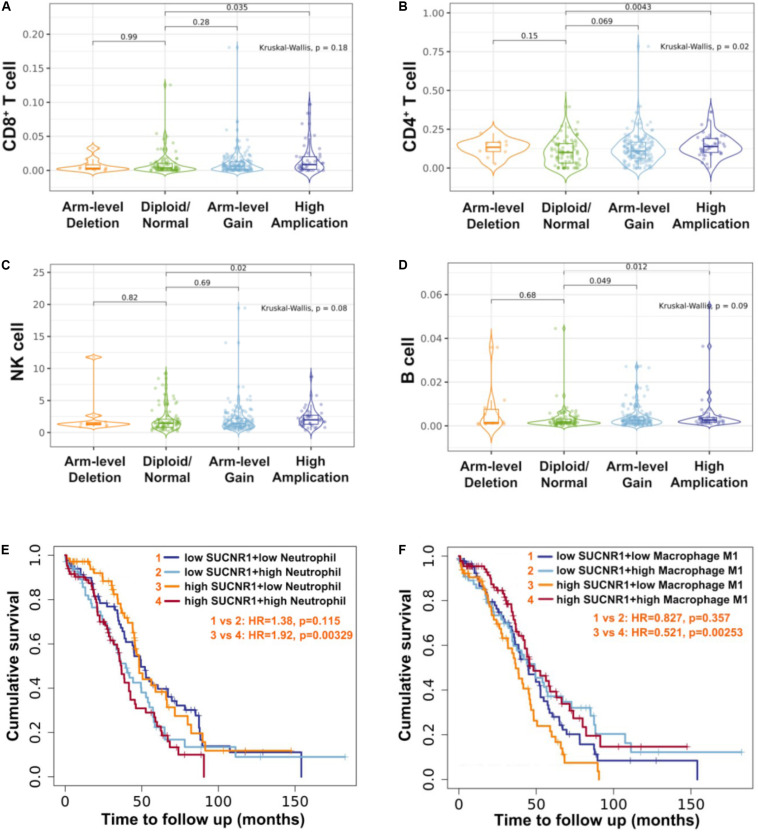
The amplification of SUCNR1 was correlated with immune infiltration. **(A–D)** Correlation of the genomic status of SUCNR1 with infiltrating levels of CD8^+^ T cells **(A)**, CD4^+^ T cells **(B)**, natural killer cells (NK) **(C)** and B cells **(D)** in ovarian cancer are available on the TIMER2.0 database. **(E)** Kaplan-Meier curves in SUCNR1/neutrophil high or low ovarian cancer patients are available on the TIMER2.0 database. **(F)** Kaplan-Meier curves in SUCNR1/M1 macrophage high or low ovarian cancer patients are available on the TIMER2.0 database.

As SUCNR1 is amplified in ovarian cancer, we further analyzed the relationship between the amplification status of SUCNR1 and immune infiltration. The TIMER2.0 database demonstrated that the immune infiltration levels of CD8^+^ T cells, CD4^+^ T cells, natural killer cells and B cells were higher in SUCNR1 amplification samples than in normal samples (*p* < 0.05) (Figures 7A–D). Additionally, high expression of SUCNR1 plus a high infiltration level of neutrophils predicted a poor survival rate (*p* = 0.00329, HR = 1.92), whereas high expression of SUCNR1 plus a high infiltration level of M1 macrophages predicted a better survival rate (*p* = 0.00253, HR = 0.521) in ovarian cancer (Figures 7E,F).

### Immune-Related Mechanisms Regulated by SUCNR1 in Ovarian Cancer

**FIGURE 8:**
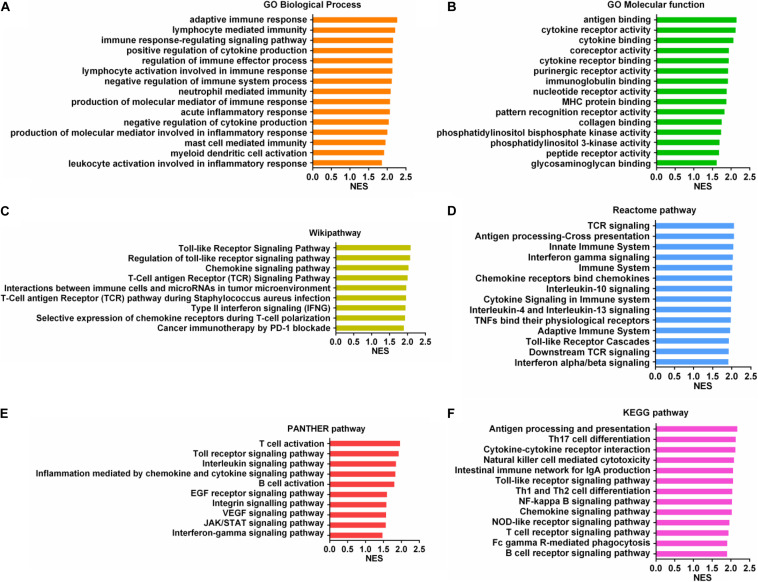
Immune-related mechanisms regulated by SUCNR1 in ovarian cancer. **(A–F)** Gene set enrichment analysis showed the correlation of SUCNR1 with GO biological process **(A)**, GO molecular function **(B)**, Wikipathway **(C)**, Reactome pathway **(D)**, PANTHER pathway **(E),** and KEGG pathway **(F)** in ovarian cancer are available on the LinkedOmics platform.

To further understand the molecular mechanism of SUCNR1 in ovarian cancer immunology, we performed a GSEA based on the Cancer Genome Atlas (TCGA) ovarian cancer RNAseq data. Gene Ontology (GO) analysis showed that SUCNR1 might be involved in adaptive immune response, lymphocyte mediated immunity, the regulation of cytokine production, regulation of the immune system process, regulation of the immune effector process, and the immune response-regulation signaling pathway (Figure 8A). Antigen binding, cytokine binding, immunoglobulin binding, MHC protein binding, and cytokine receptor activity were also potential biological roles for SUCNR1 (Figure 8B). In addition, a high expression of SUCNR1 was positively correlated with various immune-related functional biological processes of ovarian cancer mapping in KEGG pathway, Reactome pathway, Wikipathway, and Panther pathway (Figures 8C–F). For example, T cell activation, interleukin signaling pathway, chemokine signaling pathway, antigen processing and presentation, natural killer cell mediated cytotoxicity, cancer immunotherapy by PD-1 blockade, adaptive immune system, and interferon alpha/beta signaling were closely associated with the expression of SUCNR1 in ovarian cancer (Figures 8C–F). Collectively, these results further indicate that SUCNR1 participates in the immune regulation of ovarian cancer.

## Discussion

The membrane receptor SUCNR1 is a ligand of succinate and its biological function is related to metabolic indications and the inflammatory program. However, the role of SUCNR1 in regulating tumor immune cell infiltration is unclear. In the current study, we reported that SUCNR1 was amplified and predicted a poor prognosis in ovarian cancer. The expression of SUCNR1 was closely correlated with immune infiltrates, diverse immune markers, and T cell exhaustion. The upregulation of SUCNR1 could affect the expression of cytokine- or chemokine-related genes. Furthermore, GSEA also revealed that various immune-related pathways might be regulated by SUCNR1. Therefore, our study highlights the immunoregulatory role of SUCNR1 in ovarian cancer.

Succinate-triggered SUCNR1 signaling affects cellular metabolism and has been documented in multiple pathological processes, including ischemia-reperfusion, renin-induced hypertension, cardiac hypertrophy, and diabetic nephropathy and inflammation (Gilissen et al., 2016; Lückmann et al., 2020). Interestingly, during mast cell development, SUCNR1 deficiency bestows mast cells with a hyperactive phenotype (Rubić-Schneider et al., 2017). Moreover, SUCNR1 can also function as an immunologic danger sensor for immature dendritic cells to enhance antigen-presenting functions required for optimal T cell activation (Rubic et al., 2008), indicating the role of SUCNR1 in regulation of immunometabolism. Although a recent study showed that succinate enhanced the invasion and migration of lung cancer cells through SUCNR1 (Wu et al., 2020), there is still little known about the role of SUCNR1 in oncogenesis. Here, the genetic and expression profiles of SUCNR1 in ovarian cancer were analyzed using the cBioPortal, GEO and TIMER databases. Our results indicate that SUCNR1 were amplified and upregulated in ovarian cancer. It is well known that cancer antigen CA125, encoded by the MUC16, is commonly elevated in the serum of women with ovarian cancer (Felder et al., 2014). We found that MUC16 mutant ovarian cancer samples had higher SUCNR1 expression than MUC16 wild-type samples. Furthermore, SUCNR1 has also been identified as a poor prognostic factor in patients with ovarian cancer. These observations suggest that SUCNR1 may be involved in the carcinogenesis of ovarian cancer.

The biological roles of SUCNR1 in ovarian cancer were identified by GSEA. It is worth noting that the high expression of SUCNR1 was closely related to a variety of immune-related processes and pathways, including cytokine and MHC protein binding, regulation of cytokine production, regulation of immune effector process, lymphocyte mediated immunity, T cell activation, interleukin signaling pathway, natural killer cell mediated cytotoxicity, and interferon alpha/beta signaling. This indicates that SUCNR1 has an immunoregulatory role in ovarian cancer.

Although SUCNR1 has been found to regulate the function of immune cells (Rubic et al., 2008; Littlewood-Evans et al., 2016; Rubić-Schneider et al., 2017; van Diepen et al., 2017; Saraiva et al., 2018; Keiran et al., 2019), it remains to determine whether SUCNR1 is involved in modulating tumor immunity. Our analysis showed for the first time that SUCNR1 was correlated with immune cell infiltration in ovarian cancer. The immunoregulatory function of SUCNR1 was supported by the following observation: SUCNR1 expression is closely related to the infiltration level of CD8^+^ T cells, CD4^+^ T cells, macrophages, B cells, neutrophils, dendritic cells, and MDSC. In addition, the expression of SUCNR1 is closely related to the gene markers of various immune cells, including Treg (FOXP3 and CCR8), Th1 (TBX21 and CXCR3), Th2 (GATA3 and STAT5A), natural killer cells (KIR3DL1, GNLY, and PRF1), M2 macrophages (CD163, VSIG4, and MS4A4A), and even TAM (CCL2, CD68, and IL10). To our knowledge, different immune cells are recruited by different chemokines and inflammatory cytokines (Zhang et al., 2019). For example, upregulation of CXCL9 or CXCL10 is associated with increased tumor infiltrating CD8^+^ T cells and natural killer cells (Humblin and Kamphorst, 2019; Kikuchi et al., 2019; Petty et al., 2019). CCL5 promotes the recruitment of FOXP3^+^ Treg cells and macrophages (Wang et al., 2017; Walens et al., 2019). Here, we found that SUCNR1 is not only related to the expression of many cytokines, chemokines, and receptors, but also involved in their regulation. These results indicate that SUCNR1 could potentially enhance the secretion of cytokines and chemokines, thereby promoting extensive infiltration of intratumoral immune cells and play an important role in regulating tumor immunity.

T cell dysfunction and exhaustion can lead to cancer immune evasion (Jiang et al., 2015; Zhang et al., 2020). In the tumor microenvironment, the expression of inhibitory immune-checkpoint molecules is always dysregulated (Anderson and Rapoport, 2018). Our results revealed that the expressions of different exhausted T cells markers, such as CD244, PD1, LAG3, CTLA4, TIGIT, HAVCR2, PTGER2, EOMES, and GZMB, were closely related to SUCNR1 expression. Additionally, the current study also demonstrated the association between the expression of SUCNR1 and more inhibitory ligands or receptors (PD-L1, PD-L2, TNFRSF14, CD80, and CD86). Therefore, it is possible that targeting SUCNR1 might be a novel immunotherapy based on immune checkpoint regulation.

There are still some limitations in our study. We draw our conclusions through correlation analysis based on several public databases, and did not conduct *in vivo* experiments to confirm the immunoregulatory role of SUCNR1 in ovarian cancer. On the other hand, the detailed mechanisms of SUCNR1 in regulating immune cell infiltration needs further study.

In summary, our bioinformatics analysis revealed that increased SUCNR1 expression is associated with a high level of immune infiltration. Furthermore, the expression of SUCNR1 was closely associated with T cell exhaustion, gene markers of various immune cells, cytokines, and chemokines. The prognostic value of SUCNR1 was also demonstrated in patients with ovarian cancer. These findings identify SUCNR1 as a novel immune regulator and could be a valuable target for ovarian cancer immunotherapy.

## Data Availability Statement

The original contributions presented in the study are included in the article/Supplementary Material, further inquiries can be directed to the corresponding authors.

## Author Contributions

QW and YY conceived the project. JZ and QZ analyzed the data. JZ, QZ, YY, and QW contributed toward the interpretation of the data. All authors wrote and approved the final manuscript.

## Conflict of Interest

The authors declare that the research was conducted in the absence of any commercial or financial relationships that could be construed as a potential conflict of interest.

## Footnotes

1 http://cis.hku.hk/TISIDB/

2 https://cistrome.shinyapps.io/timer/

3 http://timer.cistrome.org/

4 http://www.cbioportal.org/

5 http://www.ncbi.nlm.nih.gov/geo/

6 http://kmplot.com/analysis/

7 http://linkedomics.org/login.php

## Supplementary Material

FIGURE S1 The expression of SUCNR1 is correlated with immune infiltration in ovarian cancer. **(A–F)** Correlation of SUCNR1 expression with infiltrating levels of CD8^+^ T cells (CIBERSORT-ABS), B cells (XCELL), effector memory CD4^+^ T cells (XCELL), memory resting CD4^+^ T cells (CIBERSORT-ABS), myeloid dendritic cells (XCELL), and macrophages (XCELL) in ovarian cancer.

FIGURE S2 The expression of SUCNR1 is correlated with gene makers of immune cell and T cell exhaustion in ovarian cancer. **(A)** Correlation of SUCNR1 expression with immune gene makers, including CD8A, CD68, CCR7, FCGR3B, PRF1, CD163, CD86, CD38, TBX21, and CD33, in ovarian cancer samples from GSE9891 (*n* = 285). **(B)** Correlation of SUCNR1 expression with T cell exhaustion markers, including IL7R, EOMES, CTLA4, CD244, CCL3, TIGIT, LAG3, PD-L1, PD-L2, and CD48, in ovarian cancer samples from GSE9891 (*n* = 285). Correlation coefficients (rho values) and *p* values were shown.

FIGURE S3 The expression of SUCNR1 is correlated with cytokines, chemokines and MHC molecules in ovarian cancer. **(A)** Correlation of SUCNR1 expression with cytokines, including IL7, IL15, IL16, IFNG, IFNGR1, IL2RA, IL21R, IRF1, IRF2, and IRF8, in ovarian cancer samples from GSE9891 (*n* = 285). **(B)** Correlation of SUCNR1 expression with chemokines and MHC molecules, including CCR1, CCR2, CCL5, CCL8, CCL18, CXCL9, CXCL11, HLA-DRA, HLA-B, and HLA-E, in ovarian cancer samples from GSE9891 (*n* = 285). Correlation coefficients (rho values) and *p* values were shown.

FIGURE S4 The expression of SUCNR1 is associated with cytokines and chemokines. **(A–D)** The expressions of differential genes (*Cxcl10*, *Cxcl12*, *Cxcl16*, *Ccl2*, *Ccl5*, *Ccl28*, *Il9*, *Il11*, *Il17B*, *Irf1*, *Irf3*, and *Irf5*) in visceral adipose tissue from *Sucnr1*^*flox/flox*^ and LysM-*Sucnr1*^–/–^ mice available at GSE120121.

FIGURE S5 The genetic profile and prognostic value of SUCNR1 in ovarian cancer. **(A)** Genetic alterations of SUCNR1 across different cancer types available at cBioPortal. **(B)** The expression of SUCNR1 in serous ovarian cancer samples (*n* = 30), endometrioid ovarian cancer samples (*n* = 13), and clear cell cancer samples (*n* = 16) from GSE39204. **(C)** The expression of SUCNR1 between wild-type and mutation status of indicated genes (TP53, PTEN, KRAS, BRCA1, BRCA2, and PIK3CA) of ovarian cancer samples available at TIMER. **(D)** Kaplan-Meier OS (overall survival) curves in SUCNR1 high and low expression ovarian cancer patients from GSE9891. **(E)** Kaplan-Meier OS and PFS (progression-free survival) curves in SUCNR1 high and low expression ovarian cancer patients with mutation status of p53 from GSE9891.

FIGURE S6 The mutant status of MUC16 related to immune infiltration in ovarian cancer. **(A–F)** Correlation of wild-type or mutant status of MUC16 with infiltrating levels of effector memory CD4^+^ T cells **(A)**, B cells **(B)**, monocytes **(C)**, myeloid dendritic cells **(D)**, macrophages **(E),** and M2 macrophages **(F)** in ovarian cancer are available on the TIMER2.0 database.
