# Supplementary figures and images for: Association Between Succinate Receptor SUCNR1 Expression and Immune Infiltrates in Ovarian Cancer
Source: Front Mol Biosci. 2020 Aug 31;7:150. doi: 10.3389/fmolb.2020.00150 (PMC7488939; doi:10.3389/fmolb.2020.00150)

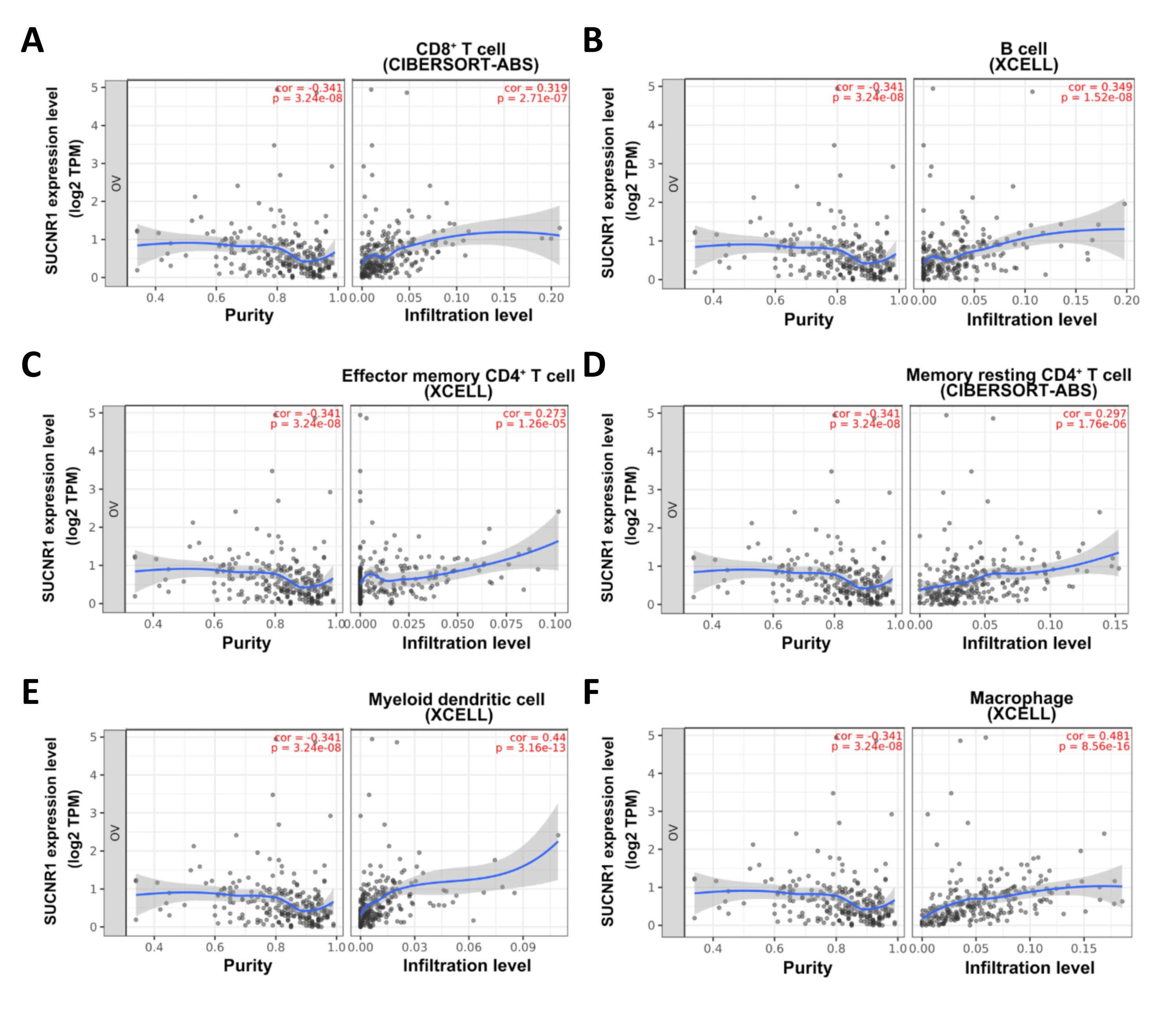

Supplement: FIGURE S1 — The expression of SUCNR1 is correlated with immune infiltration in ovarian cancer. (A–F) Correlation of SUCNR1 expression with infiltrating levels of CD8+ T cells (CIBERSORT-ABS), B cells (XCELL), effector memory CD4+ T cells (XCELL), memory resting CD4+ T cells (CIBERSORT-ABS), myeloid dendritic cells (XCELL), and macrophages (XCELL) in ovarian cancer. [file Image_1.TIF]

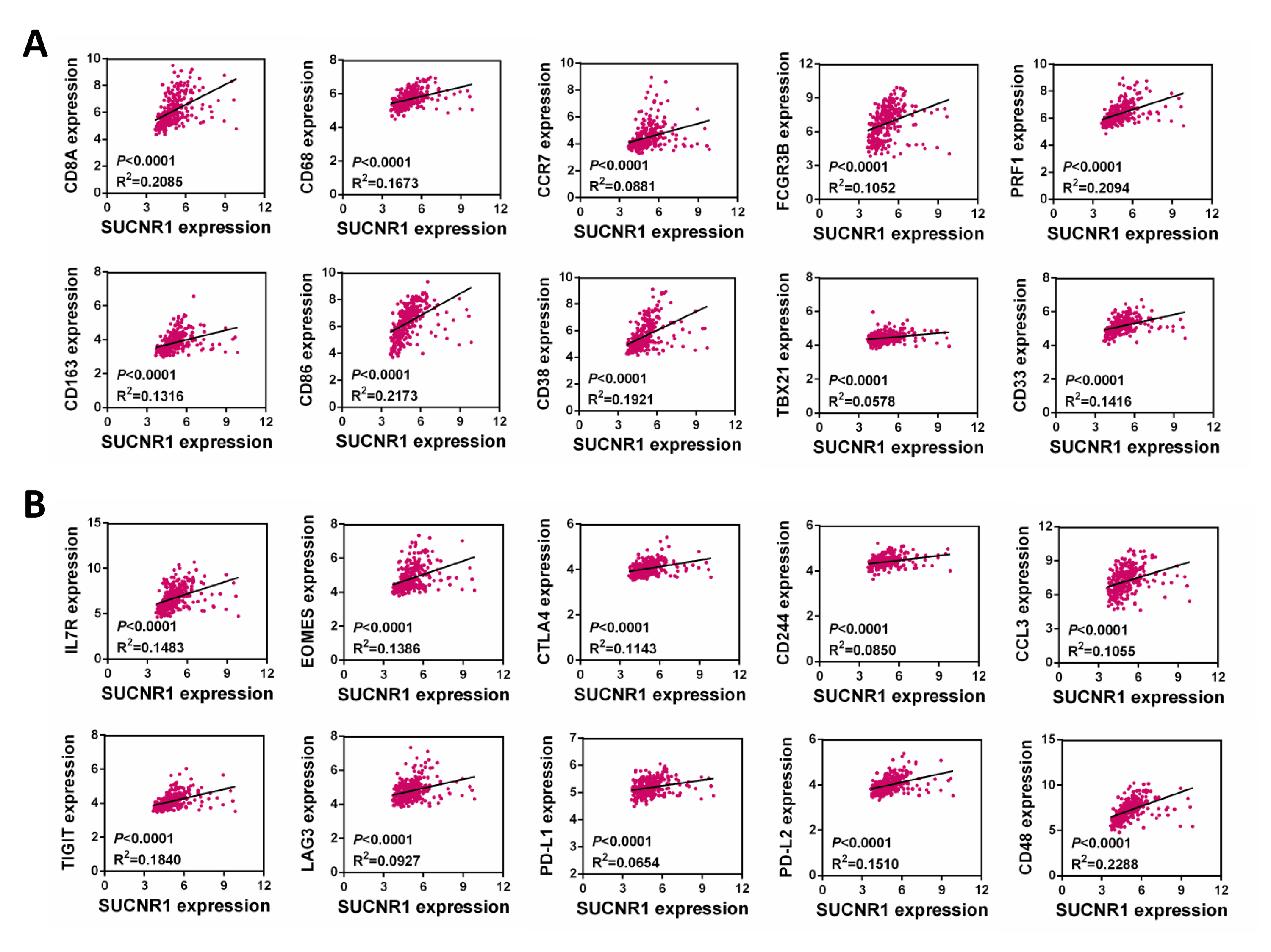

Supplement: FIGURE S2 — The expression of SUCNR1 is correlated with gene makers of immune cell and T cell exhaustion in ovarian cancer. (A) Correlation of SUCNR1 expression with immune gene makers, including CD8A, CD68, CCR7, FCGR3B, PRF1, CD163, CD86, CD38, TBX21, and CD33, in ovarian cancer samples from GSE9891 (n = 285). (B) Correlation of SUCNR1 expression with T cell exhaustion markers, including IL7R, EOMES, CTLA4, CD244, CCL3, TIGIT, LAG3, PD-L1, PD-L2, and CD48, in ovarian cancer samples from GSE9891 (n = 285). Correlation coefficients (rho values) and p values were shown. [file Image_2.TIF]

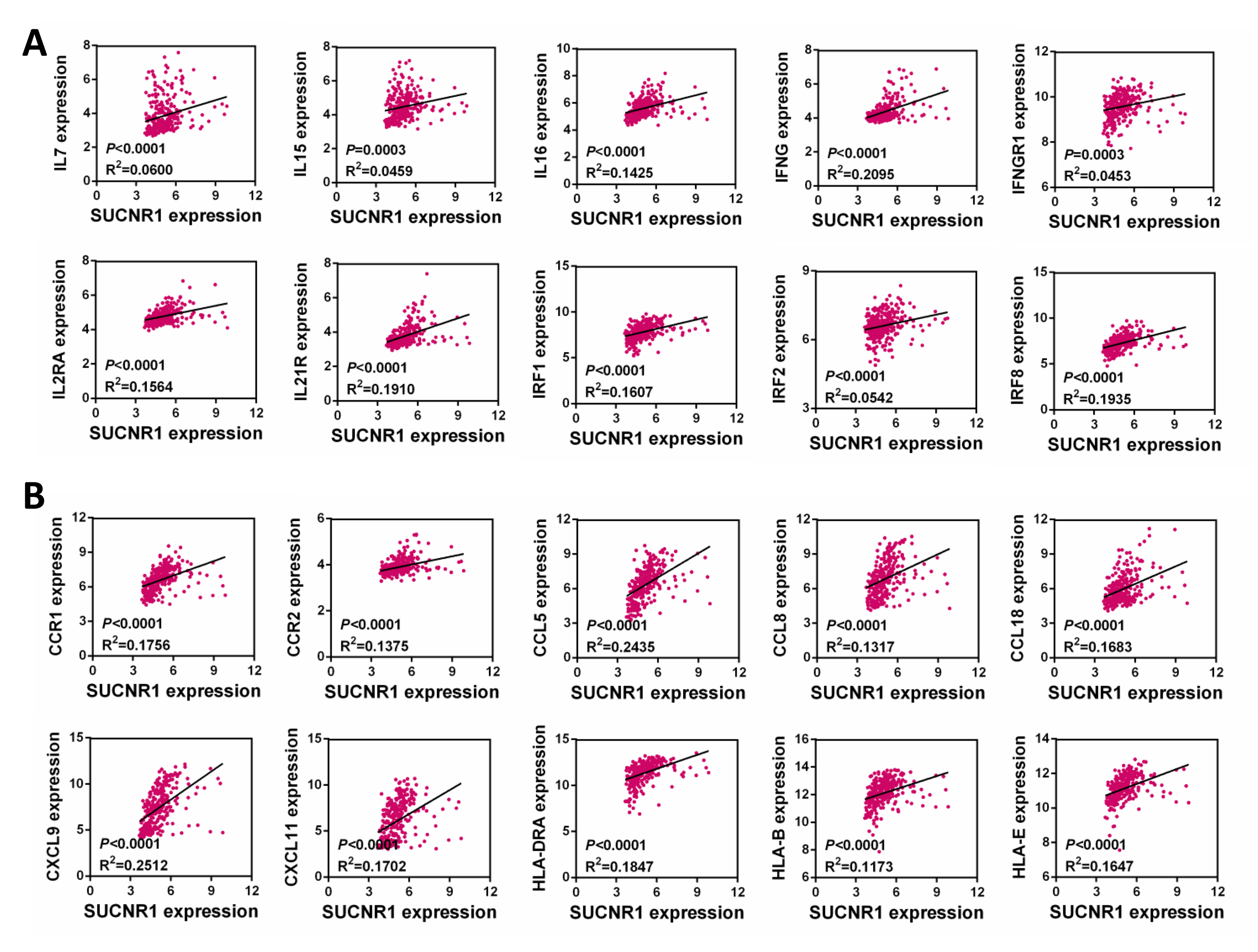

Supplement: FIGURE S3 — The expression of SUCNR1 is correlated with cytokines, chemokines and MHC molecules in ovarian cancer. (A) Correlation of SUCNR1 expression with cytokines, including IL7, IL15, IL16, IFNG, IFNGR1, IL2RA, IL21R, IRF1, IRF2, and IRF8, in ovarian cancer samples from GSE9891 (n = 285). (B) Correlation of SUCNR1 expression with chemokines and MHC molecules, including CCR1, CCR2, CCL5, CCL8, CCL18, CXCL9, CXCL11, HLA-DRA, HLA-B, and HLA-E, in ovarian cancer samples from GSE9891 (n = 285). Correlation coefficients (rho values) and p values were shown. [file Image_3.TIF]

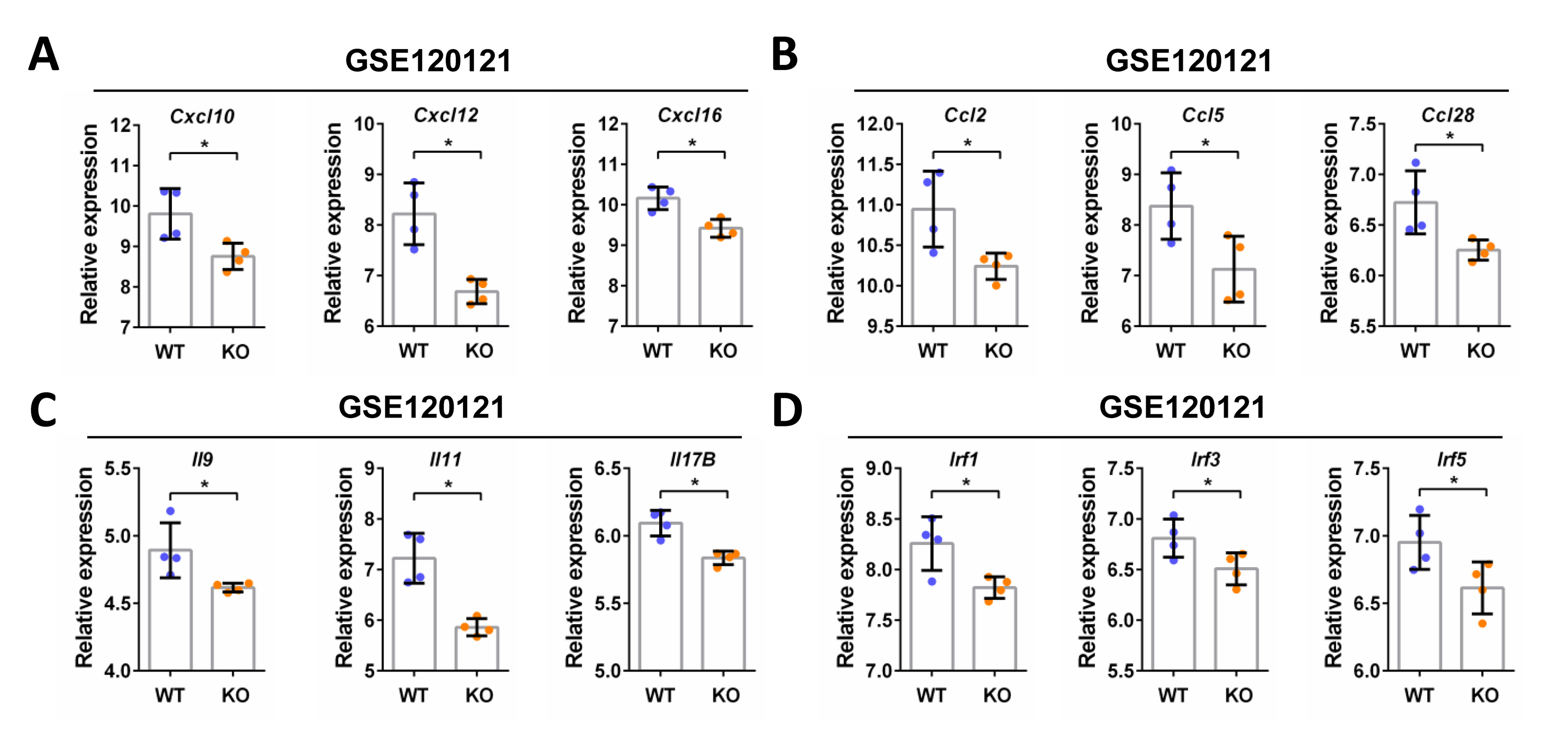

Supplement: FIGURE S4 — The expression of SUCNR1 is associated with cytokines and chemokines. (A–D) The expressions of differential genes (Cxcl10, Cxcl12, Cxcl16, Ccl2, Ccl5, Ccl28, Il9, Il11, Il17B, Irf1, Irf3, and Irf5) in visceral adipose tissue from Sucnr1flox/flox and LysM-Sucnr1–/– mice available at GSE120121. [file Image_4.TIF]

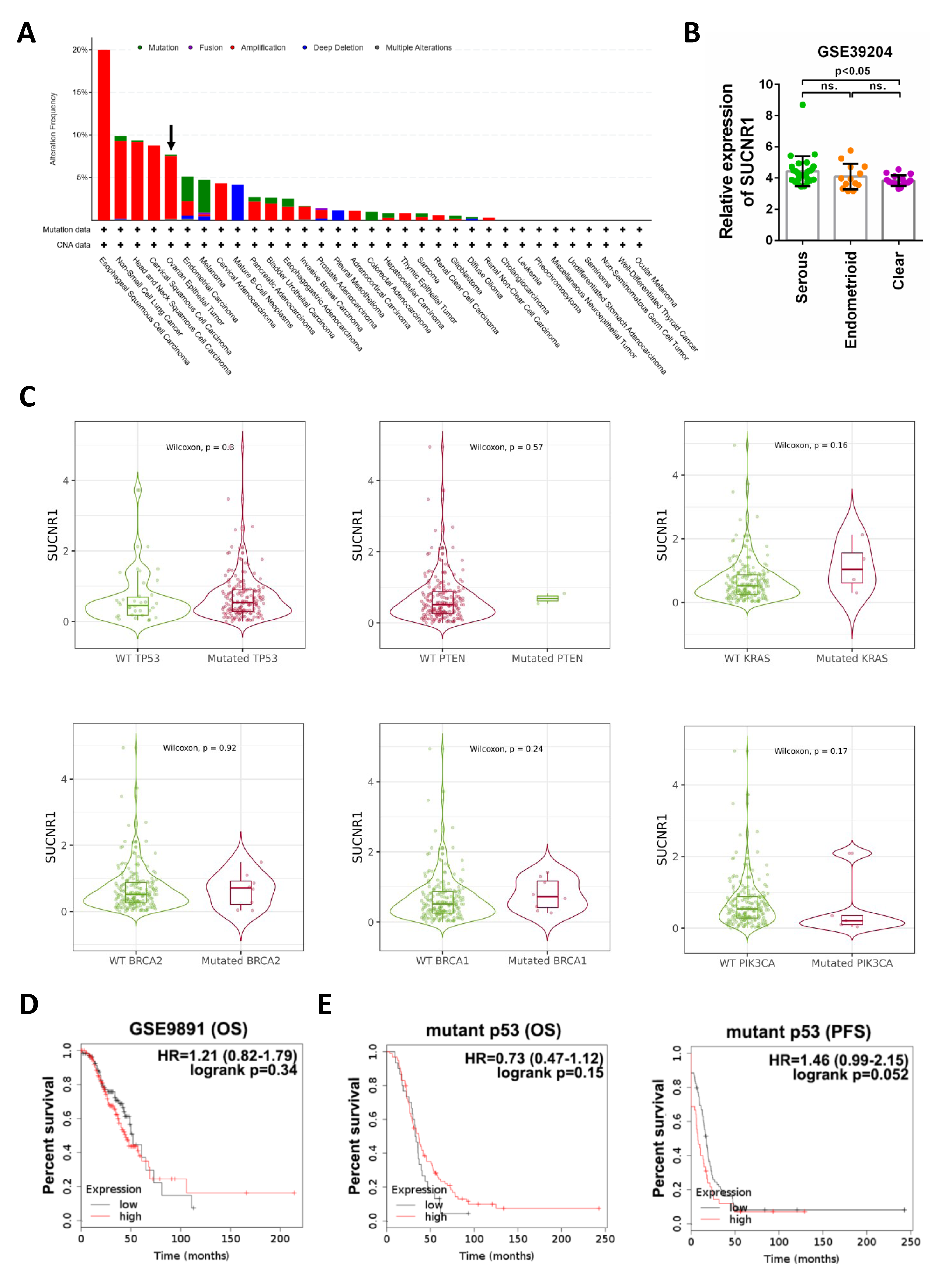

Supplement: FIGURE S5 — The genetic profile and prognostic value of SUCNR1 in ovarian cancer. (A) Genetic alterations of SUCNR1 across different cancer types available at cBioPortal. (B) The expression of SUCNR1 in serous ovarian cancer samples (n = 30), endometrioid ovarian cancer samples (n = 13), and clear cell cancer samples (n = 16) from GSE39204. (C) The expression of SUCNR1 between wild-type and mutation status of indicated genes (TP53, PTEN, KRAS, BRCA1, BRCA2, and PIK3CA) of ovarian cancer samples available at TIMER. (D) Kaplan-Meier OS (overall survival) curves in SUCNR1 high and low expression ovarian cancer patients from GSE9891. (E) Kaplan-Meier OS and PFS (progression-free survival) curves in SUCNR1 high and low expression ovarian cancer patients with mutation status of p53 from GSE9891. [file Image_5.TIF]

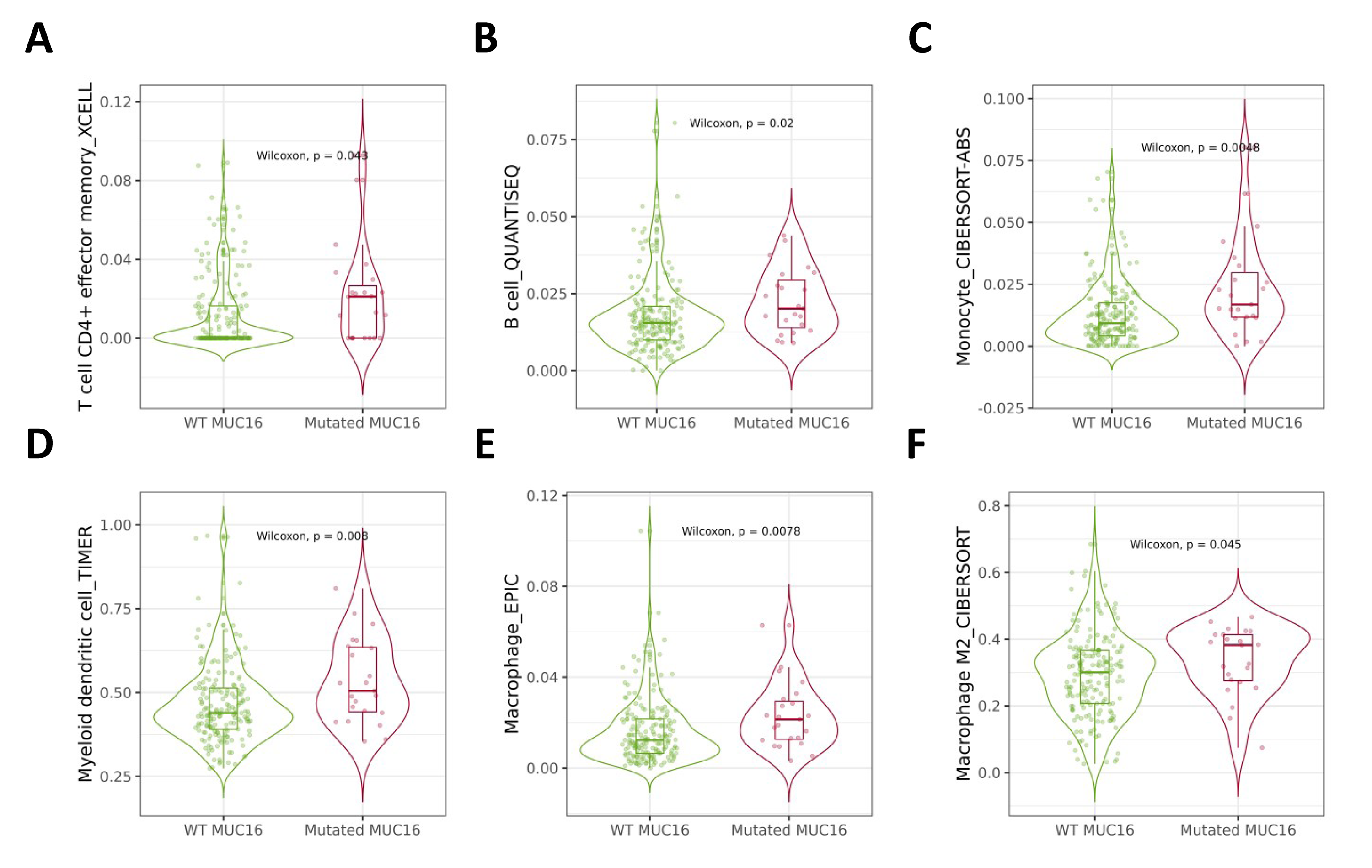

Supplement: FIGURE S6 — The mutant status of MUC16 related to immune infiltration in ovarian cancer. (A–F) Correlation of wild-type or mutant status of MUC16 with infiltrating levels of effector memory CD4+ T cells (A), B cells (B), monocytes (C), myeloid dendritic cells (D), macrophages (E), and M2 macrophages (F) in ovarian cancer are available on the TIMER2.0 database. [file Image_6.TIF]
